# Evaluation of the patient-accompanying app “alley ortho companion” for patients with osteoarthritis of the knee and hip: study protocol for a randomized controlled multi-center trial

**DOI:** 10.1186/s13063-022-06662-6

**Published:** 2022-08-29

**Authors:** André Strahl, Heiko Graichen, Holger Haas, Robert Hube, Carsten Perka, Tim Rolvien, Jan Hubert

**Affiliations:** 1 Division of Orthopaedics, Department of Trauma and Orthopaedic Surgery, University Medical Center Hamburg-Eppendorf, Martinistr. 52, 20246 Hamburg, Germany; 2 Department for Arthroplasty, Asklepios Orthopaedic Hospital Lindenlohe, Lindenlohe 18, 92421 Schwandorf, Germany; 3 Community Hospital Bonn, House St. Petrus, Center of Orthopaedics and Trauma Surgery, Bonner Talweg 4-6, 53113 Bonn, Germany; 4 OCM Clinic Munich, Steinerstr. 6, 81369 Munich, Germany; 5 Center for Musculoskeletal Surgery, Charité-Universitätsmedizin Berlin, corporate member of Freie Universität Berlin, Humboldt-Universität zu Berlin, and Berlin Institute of Health, Schumannstr. 20, 10117 Berlin, Germany

**Keywords:** Osteoarthritis, Total hip arthroplasty, Total knee arthroplasty, eHealth application, Health literacy, Randomized controlled trial

## Abstract

**Background:**

Osteoarthritis (OA) is one of the most common disabilities in the elderly. When conservative management fails, total joint arthroplasty (TJA) is the treatment of choice for end-stage OA. Since quality and durability of implants has steadily improved, pre -and postsurgical processes moved into the focus of research. Hence, eHealth approaches offer an opportunity to provide a more available continuity of care. Regarding individualized pre-, peri-, and postsurgical stages, eHealth is expected to improve patient engagement, self-care, and outcomes across the surgical pathway. Aim of this study is to evaluate the effectiveness of the eHealth application “alley” as an adjuvant intervention to TJA. The app provides comprehensive information to empower patient with hip or knee OA to prepare and accompany them for their TJA surgery. Our primary hypothesis is that the pre- and postoperative adjuvant use of the eHealth application “alley” (intervention group, IG) leads to improved functional outcome.

**Methods:**

Prospective, randomized, controlled, multi-center trial including *n* = 200 patients diagnosed with hip and *n* = 200 patients with knee OA (*n* = 200) scheduled for TJA. Patients of both groups will be randomly assigned to one of two study arms. Patients in the intervention group will receive access to the functions of the “alley” app. The app presents informative (e.g., information about osteoarthritis), organizational (e.g., information about medical rehabilitation), and emotional/empowerment (e.g., information about the relationship between mood and pain) content. Patients evaluate their condition and functional level by means of standardized digitally questionnaires. Patients in the control group will not receive any functions of the app. Assessments will be performed at baseline before, 10 days after, 1 months after, 3 months after, 6 months after, and 12 months after TJA. Primary outcome is change from baseline measured by the Hip Osteoarthritis Outcome Score or Knee injury and Osteoarthritis Outcome Score 3 months after TJA. The statistical analysis (*t*-test for independent variables with effect size Cohen’s *d*) is performed separately for patients with TKA and THA.

**Discussion:**

Overall, the study aims to improve the understanding of the benefits of eHealth applications in the treatment of elderly patients with knee or hip arthroplasty. The approach is novel since a health care companion is combined with a digital information platform enabling direct and continuous feedback from the patients to the therapeutic treatment team. As the study investigate the effectiveness under everyday conditions, it is not feasible to control whether the patients in the IG read the educational information of the app respectively the control group consume additional information from other sources. However, this increases the external validity of the study if significant effects for the app can be demonstrated.

**Trial registration:**

German Clinical Trials Register: DRKS00025608. Registered on 21 June 2021.

## Administrative information

Note: The numbers in curly brackets in this protocol refer to SPIRIT checklist item numbers. The order of the items has been modified to group similar items (see http://www.equator-network.org/reporting-guidelines/spirit-2013-statement-defining-standard-protocol-items-for-clinical-trials/).

**Table:** 

Title {1}	Evaluation of the patient-accompanying App “alley ortho companion” for patients with osteoarthritis of the knee and hip: Study protocol for a randomized controlled multi-center trial
Trial registration {2a and 2b}.	German Clinical Trials Register (https://www.drks.de/): registration code DRKS00025608
Protocol version {3}	Ethical approved study protocol Date: 12/5/2021 (version 2)
Funding {4}	VBMC ValueBasedManagedCare GmbH Schanzenstraße 30 51063 Köln The Funder provides financial support for expenses and is operator of the app to be evaluated
Author details {5a}	Dr. André Strahl, M.Sc. Division of Orthopaedics, Department of Trauma and Orthopaedic Surgery University Medical Center Hamburg-Eppendorf Martinistr. 52, D-20246 Hamburg, Germany Prof. Dr. Heiko Graichen Department for Arthroplasty Asklepios Orthopaedic Hospital Lindenlohe Lindenlohe 18, D-92421 Schwandorf, Germany Dr. Holger Haas Community Hospital Bonn House St. Petrus Center of Orthopaedics and Trauma Surgery Bonner Talweg 4-6, D-53113 Bonn, Germany Prof. Dr. Robert Hube OCM Clinic Munich Steinerstr. 6, D-81369 Munich, Germany Prof. Dr. Carsten Perka Center for Musculoskeletal Surgery Charité-Universitätsmedizin Berlin, corporate member of Freie Universität Berlin, Humboldt-Universität zu Berlin, and Berlin Institute of Health Schumannstr. 20, D-10117 Berlin, Germany PD Dr. Dr. Tim Rolvien, MBA, Division of Orthopaedics, Department of Trauma and Orthopaedic Surgery University Medical Center Hamburg-Eppendorf Martinistr. 52, D-20246 Hamburg, Germany PD Dr. Jan Hubert Division of Orthopaedics, Department of Trauma and Orthopaedic Surgery University Medical Center Hamburg-Eppendorf Martinistr. 52, D-20246 Hamburg, Germany
Name and contact information for the trial sponsor {5b}	VBMC ValueBasedManagedCare GmbH Schanzenstraße 30 51063 Köln
Role of sponsor {5c}	The sponsor and funding body had no influence on the design of the study, the data collection and analysis, the data interpretation or in writing the manuscript.

## Introduction

### Background and rationale {6a}

Osteoarthritis (OA) is one of the most common disabilities in the elderly. With a lifetime risk of 25% in the population aged 85 years, symptomatic hip OA represents one of the most prevalent diseases [1]. Similarly, the prevalence of knee OA increases with age and can be detected in up to 44% in subjects older than 80 years [2]. OA is a heterogeneous disease evoked by several causes, including local and systemic, genetic, and environmental factors, and is consequently difficult to prevent [3, 4]. When conservative management fails, total joint arthroplasty (TJA) is the treatment of choice for end-stage OA to reliably relieve pain and restore joint function [5, 6]. TJA is considered to be one of the most successful surgical procedures, while it has been shown as a cost-effective intervention for patients who do not benefit from conservative approaches [7]. Worldwide, more than 1 million TJAs are implanted annually [8], and it is expected that in future the number of surgeries will further increase due to demographic changes [3, 9].

Since the quality and durability of implants has steadily improved over decades, the pre -and postsurgical processes moved into the focus of research in order to achieve potential improvements. For instance, the optimal care after hospitalization has not yet been determined and shows a broad variety of treatment [10]. From diagnosis, therapy and rehabilitation to aftercare, patients have intensive contact with various actors of the health care system. This patient’s “journey” often extends over years and entails several loops within the health sector, resulting in high costs and diverse short and long-term results. Process optimization pathways in health care represent a possibility with great economic leverage. Moreover, these improvements may enhance process outcomes and thereby enable long-term savings. Current literature identified options for improving specific parts of the patient`s journey, e.g., patient satisfaction as quality indicator of healthcare or patient adherence [11, 12]. Further studies specified patient education as distinct option for optimizing patient pathways, while preoperative education and knowledge showed positive effects regarding adverse events, cost-effectiveness, and pain and function after TJA [13] as well as improved outcomes and reduced hospital stay [14, 15]. In order to achieve an implementation in clinical routine, eHealth approaches are currently being deployed, e.g., to address patient education with regard to TJA [16]. EHealth, defined as “the use of information and communication technologies for health” by the WHO [17], offers an opportunity for health professionals to provide an enhanced and more available continuity of care to serve the patients individual needs [18]. Digital interventions can be constantly updated, have the potential to increase patient engagement, enhance patient recovery, and reduce potential postoperative complications [16]. In addition, eHealth may improve cost-effectiveness as patients can receive time- and space-independent support before and after treatment [19, 20, 21]. Regarding individualized pre-, peri-, and postsurgical stages, eHealth is expected to improve patient engagement, self-care, and outcomes across the surgical pathway [18].

### Objectives {7}

Aim of the study is to evaluate the effectiveness of the eHealth application “alley” as an adjuvant intervention to total joint arthroplasty (TJA) as current gold standard of treatment in patients with knee and hip OA scheduled for surgery. In the presented study design, these two indications will be investigated independently of each other. Nevertheless, for both patients’ groups data collection is carried out via “alley” application. Accordingly, the primary outcome is change from baseline in the functional status measured with the total scores of the Hip Osteoarthritis Outcome Score (HOOS) for patients scheduled for total hip arthroplasty (THA) and the Knee injury and Osteoarthritis Outcome Score (KOOS) for patients scheduled for total knee arthroplasty (TKA).

The primary hypothesis is that the pre- and postoperative adjuvant use of the eHealth application “alley” (intervention group, IG) leads to improved functional outcome 3 months after TJA in patients with knee or hip OA compared to their equivalent control group (CG) that did not use the application. In this research, the overall effect of the application as complex intervention will be evaluated as standalone entity. Individual sub-questions on the subject of health literacy or in which format information should be administered to patients are not evaluated. Our secondary hypotheses are that the eHealth application will further improve pain, quality of life, depression and anxiety, optimistic mood, and risk of falling.

### Trial design {8}

The trial is planned as a prospective, randomized, controlled, non-blinded multi-center study to compare “alley” with standard treatment with scope of a superiority study design in two independent indications. Two hundred patients scheduled for THA and 200 patients scheduled for TKA will be independently randomly assigned to the IG or CG. Assessments will be performed at baseline before TJA (*t*_0_), 10 days after TJA (*t*_1_), 1 months after TJA (*t*_2_), 3 months after TJA (*t*_3_), 6 months after TJA (*t*_4_), and 12 months after TJA (*t*_5_).

## Methods: participants, interventions, and outcomes

### Study setting {9}

The study will be conducted in eight clinical sites in Germany. A total of two academic hospitals and six community clinics participate in the study. The full list of these clinics can be obtained via German Clinical Trials Register (https://www.drks.de/) under the registration code DRKS00025608.

### Eligibility criteria {10}

Patients presenting at one of the study sites with the indication for knee or hip replacement who are scheduled for TJA will be screened for inclusion and exclusion criteria. The screening is usually performed by the physician in charge during the consultation. Study inclusion is permitted with a signed informed consent form. Inclusion and exclusion criteria are displayed in Table 1.

**Table 1:** Inclusion and exclusion criteria of the alley app trial

**Inclusion criteria**	
1. Scheduled hip or knee arthroplasty 2. (OPS code 5-820/5-822)	
3. Both gender the ages older than 18 years	
4. Ownership of a smartphone or internet-enabled device (e.g., tablet)	
**Exclusion criteria**	
1. Knee or hip arthroplasty due to emergency (e.g., after a fall)	
2. Knee or hip arthroplasty due to revision surgery	
3. Level 3 care degree or higher according to the German long-term care insurance	
4. Cognitive impairment of any kind (according to the assessment of the attending physicians) that prevents the proper use of the app	
5. Neurological disorder (e.g., Parkinson’s disease, MS, dementia) (according to the assessment of the treating doctors) that prevents the proper use of the app	
6. Severe psychiatric disorder that prevents the proper use of the app.	
7. Patient’s age younger than 18 years	
8. Insufficient knowledge of German language to use the app	
9. No ownership of a smartphone or internet-enabled device	
10. Women in pregnancy	
11. Women during lactation	

### Who will take informed consent? {26a}

During the first ambulatory visit, patients are verbally informed about the study by the attending physician in conjunction with a written patient information. At this point, the patients are already screened and assigned to the CG or IG by means of randomization. After this visit, patients have time to consider participation until the next mandatory pre-stationary consultation (1 to 2 weeks before surgery). In case of study participation, the group allocation remains. If a decision is made against participation, the allocation is canceled.

### Additional consent provisions for collection and use of participant data and biological specimens {26b}

Not applicable. No biological specimens will be obtained.

### Interventions

#### Explanation for the choice of comparators {6b}

Patients in the CG also have an indication for knee or hip replacement and receive the current state-of-the-art, gold standard therapeutic intervention (TJA). The comparator group differs from the IG in the fact that patients cannot use the functions of the “alley” application. Since alley is an accompanying adjuvant eHealth intervention, this type of CG is well-suited to directly compare the effects of the app solely. Nevertheless, the (non-functional) application must be installed on a smartphone or an internet-enabled mobile device as it is used for the questionnaire survey and data entry.

#### Intervention description {11a}

The adjuvant use of the “alley” eHealth application in addition to TJA is defined as IG. The application is hosted by the VBMC ValueBasedManagedCare GmbH and is offered for Android and Apple mobile devices. The application is intended to prepare and accompany hip or knee arthroplasty. The application contains educational content, which is displayed on the patient’s smart device according to their actual individual needs within the healthcare system. The program and its contents were systematically developed by a multi-professional team consisting of medical physicians, psychologists, health scientists, economists, and computer scientists. The development was realized in a multi-stage process with the involvement of all project partners and external consultants. Development was based on existing guidelines and evidence-based materials for patient information. Didactically, text-based information modules were elaborated to guide patients through the health care system before and after surgery. The app provides comprehensive information to empower the patients. Patients receive push notifications on their mobile phones at regular intervals before and after surgery. The content of patient education is structured into three dimensions: (1) informative (information about daily life with osteoarthritis, origin of pain, treatment options, surgical procedures, daily life with joint replacement), (2) organizational (information about important contact persons after surgery, medical rehabilitation proposal, medical and non-medical remedies and aids), and (3) emotional/empowerment (information about rehabilitation targets, relationship between mood and pain, new habits, managing daily challenges). In summary, patients receive information about the disease, its treatment and aftercare as well as strategies for actively coping with the disease and its implications. These include cognitive and behavioral strategies for emotional relief, well-being, and social support. The app further provides information about which medical documents should be brought to the attending physician and which topics may be relevant to ask during a doctor’s consultation.

In the app, patients have the possibility to evaluate their condition and functional level by means of standardized medically validated questionnaires. Data on pain and functioning can continuously be entered by patients as personal monitoring.

In the IG, these patient’s data can be displayed on the physician’s web-based dashboard in the hospital to provide in-depth patient-reported information during operational reconnaissance. In line with the general purpose, the collected information is to be used in a supportive and accompanying manner by the patient and service providers involved to improve quality and optimize diagnostics and therapy. The aim of the application is to guide patients with osteoarthritis of the hip a knee through the health care system, to provide support for these patients, and to provide the attending physician with continuous information about the patient’s health. The patient reported outcome questionnaires implemented in this study are not only used as part of it but represent an integral part of the application enabling patients to report their actual condition to their treating physician at any time.-The app intervention therefore promotes individualized medicine. In the context of the study, the use of the web-based dashboard by physicians is optional.

#### Criteria for discontinuing or modifying allocated interventions {11b}

A discontinuing or modifying allocated intervention during the study period is not foreseen. The programming code of the application cannot be changed or adapted during the study. External conditions, e.g., arthroplasty joint revisions, have no influence on the use of the app. On the contrary, postoperative complications should be recorded descriptively with the application at follow-up.

### Strategies to improve adherence to interventions {11c}

Patients are reminded via push notification when new content is available to read. The notifications remain in the alley application until the corresponding information article has been read. In addition, each patient receives 150 EUR after the questionnaire has been completed 3 months after surgery.

### Relevant concomitant care permitted or prohibited during the trial {11d}

The study is designed within the scope of health services research to evaluate alley in the real environment. Accordingly, all additional interventions, by the patient, e.g., physical therapy, additional pain medication are permitted and are surveyed.

### Provisions for post-trial care {30}

Patients can continue to use the app for self-monitoring after the trial. The patients who were initially in the CG get access to the educational materials in the alley application after 12 months, when the trial is completed.

### Outcomes {12}

Standardized and self-constructed questionnaires are applied to operationalize the target criteria. After registration with alley, all patients undergo an onboarding process, in which patients complete the medical questionnaires. In total, patients are surveyed at six measurement time points: Baseline before the surgery (*t*_0_) and 10 days after surgery (*t*_1_) as well as 1 (*t*_2_), 3 (*t*_3_), 6 (*t*_4_), and 12 months (*t*_5_) after TJA.

The primary outcome is change from baseline in functional status measured by the Hip Osteoarthritis Outcome Score (HOOS) or Knee injury and Osteoarthritis Outcome Score (KOOS) 3 months after TJA. The statistical analysis will be carried out separately for the two medical indications. Further secondary endpoints of the study are depression, anxiety, physiological, mental, and social health, pain, risk of falling, dispositional optimism and pessimism, patients’ expectation, post-operative complications, satisfaction with treatment, perceived knowledge of the patient about the disease, and health literacy. The secondary endpoints will also be analyzed separately for patients with hip and knee complaints. An overview of the instruments and measurement times is given in Table 2.

**Table 2:** Target criteria, assessment instruments, and measurement time points

Target criteria	Assessment instruments	Measurement time points					
		***t***_***0***_	***t***_***1***_	***t***_***2***_	***t***_***3***_	***t***_***4***_	***t***_***5***_
**Primary outcome**							
Joint functioning	Patients with hip replacement: Hip Osteoarthritis Outcome Score (HOOS [22];)	X	X	X	X	X	X
	Patients with knee replacement: Knee injury and Osteoarthritis Outcome Score (KOOS [23];)	X	X	X	X	X	X
**Secondary outcomes**							
Pain	Visual analog scale (VAS)	X	X	X	X	X	X
Depression and anxiety	Depression, anxiety and stress scale (DASS [24];)	X	X	X	X	X	X
Physiological, mental, and social health	Patient-Reported Outcomes Measurement Information System (PROMIS®-29 [25];)	X	X	X	X	X	X
Patients’ expectations	Credibility/expectancy questionnaire (adapted from [26])	X	X	X	X	X	X
Patient satisfaction	Patients’ Experience Questionnaire (PEQ [27];)			X	X		
Dispositional optimism and pessimism	Life-Orientation-Test (LOT-R [28];)	X			X	X	X
Risk of falling	Self-constructed questionnaire	X			X	X	X
Post-operative complications	Self-constructed questionnaire		X	X	X	X	X
Knowledge about the disease (patients’ health literacy)	Self-constructed questionnaire	X	X				
Knowledge about postoperative behavior (patients’ health literacy)	Self-constructed questionnaire	X	X				
Perceived knowledge of the patient about the disease (Physician questionnaire)	Self-constructed questionnaire	X					
Sociodemographic variables	ICHOM standard set for hip- and knee osteoarthritis (International Consortium for Health Outcomes Measurement)	X					

### Participant timeline {13}

**Fig. 1:**
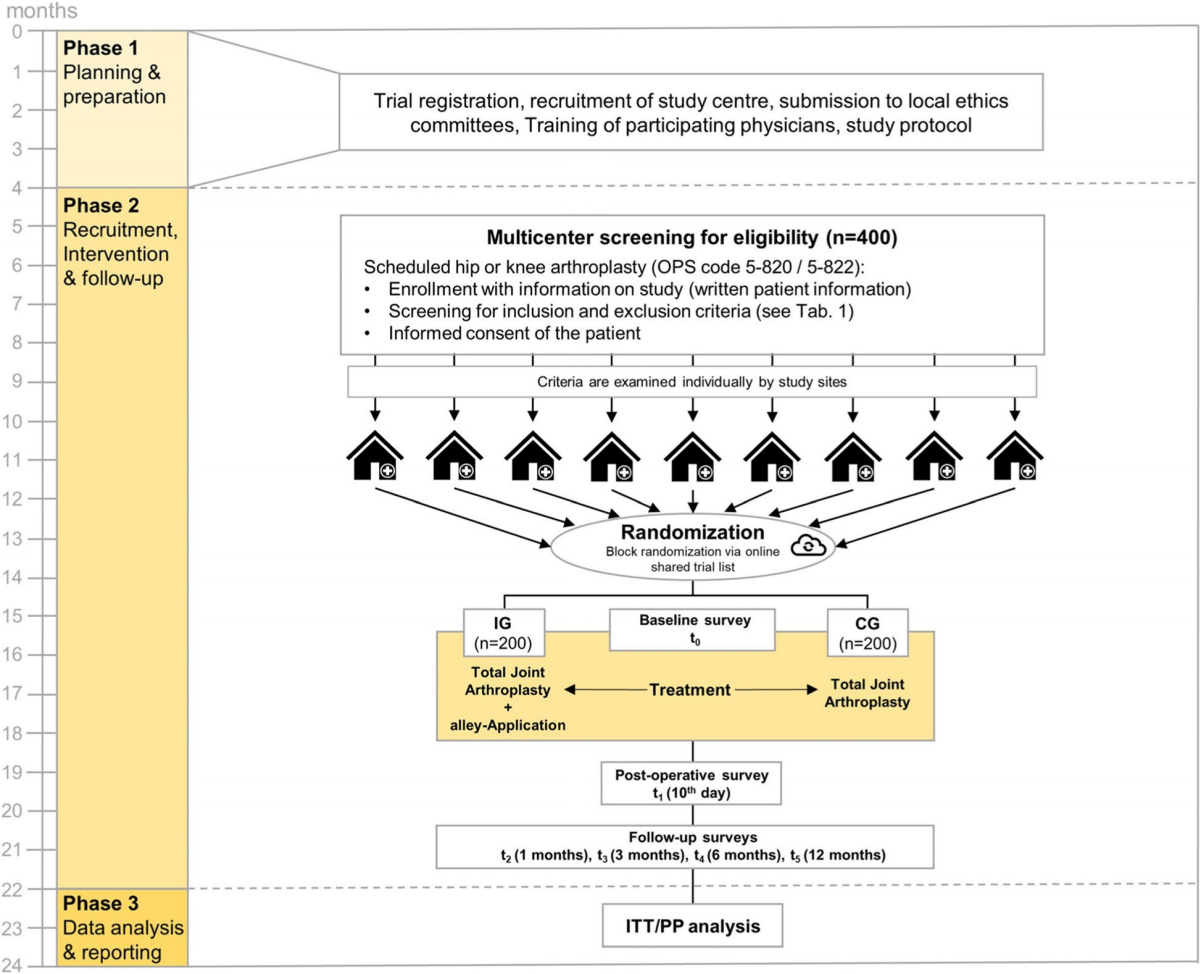
Trial design

An overview over the participant timeline is provided in the flowchart in Fig. 1 and supplemented with the questionnaires to be completed in Table 2. The patients complete various questionnaires at six measurement points. This results in a study time expenditure of approx. 180 min for the patients.

### Sample size {14}

The sample size was calculated considering the expected effect size, power, measurement time point, and design effect and was performed with the open access program G*POWER [29]. The statistical analysis and corresponding sample size calculation are conducted separately for hip and knee patients.

#### Sample size calculation for patients with osteoarthritis of the hip

The sample size calculation for patients with osteoarthritis of the hip is based on an average change score of 9 points with a standard deviation of ± 15 points in the total score of HOOS observed in other intervention studies after three months [30, 31]. Based on this data available, an effect of 0.6 can be assumed. With a standard α-risk of 0.05 and a statistical power of 0.8 (1 – β), a sample size of *n* = 61 patients in each group is required to obtain significant differences in Student’s *t*-test for independent variables. This number of cases will be enlarged to consider any potential data missing. Due to observed dropout rates of up to 20% [32], exclusion of patients due to errors in data entry (4%) [33], an approximate across-the-board increase due to the risk of complications due to corona the number of patients to be included is increased to achieve the required sample size at the time of measurement 3 months post-surgery. Therefore, *n* = 100 participants and *n* = 100 controls were supposed to be recruited for the THA evaluation group.

#### Sample size calculation for patients with osteoarthritis of the knee

The required sample size for patients with osteoarthritis of the knee is also calculated using data from the literature [30, 34, 35]. Similar to the results on the hip, we derived an average change score of 9 points with a standard deviation of ± 15 points in the total score of the KOOS yielding to an expected effect size of 0.6. With an α-risk of 0.05 and a statistical power of 0.8 (1 – β), a sample size of *n* = 61 patients in each group is required to obtain significant differences in Student’s *t*-test for independent variables. The number of required cases is further expanded to consider any missing. I summary, *n* = 100 patients and *n* = 100 controls were supposed to be recruited for the TKA evaluation group.

Therefore, the total number of patients to be included is *n* = 400.

### Recruitment {15}

Recruitment is performed by medical consultants in each of the eight study locations. Immediately after surgery indication for total hip/knee arthroplasty (THA/TKA), patients will be informed about the study and receive further information material. The patients are addressed directly by the attending physician, which increases compliance to study participation. Each study site recruits both patients with knee or hip problems and allocate these patients to the corresponding group based on the randomization sequence.

### Assignment of interventions: allocation

#### Sequence generation {16a}

A 1:1 randomization has been conducted, assigning patients to either CG or IG for THA or TKA, respectively. For this purpose, two randomization lists have been created using Excel random number generator, creating separate lists for THA and TKA. These lists contained a predefined identification code for each indication, which was randomly assigned to the IG or CG. If patients agreed to participate, they were assigned to this list and thus received a unique identification code. This personal code serves as login for the “alley” application. As the randomization list was a shared online document for all study sites, simultaneous double submissions had to be avoided. For this reason, block randomization has been used for blocks of 50 patients. This means that each of the eight study sites is allocated to a fixed slot of 50 patients on the randomization list. During patient recruitment, these slots are successively filled. If more than 50 patients are to be recruited, new slots can be released at the end of the list. The randomized allocation of patients to either IG or CG allows minimizing the inclusion of patients with strong affinity for using digital applications in the IG. Randomization will ensure an equal distribution of patients.

#### Concealment mechanism {16b}

The randomization codes are accessible to all study sites in a shared trial list. The list is located in a secure “container” on the internet that can be accessed after registration (www.tresorit.de). Access is granted after two-factor authentication. No personal patient data are stored in the online directory. The list contains study ID, group affiliation, a unique dial-in pin for the alley application, the study arm (hip or knee), a selection field for marking the ID used, and a selection field for marking the hospital. The assignment of the allocated IDs to corresponding patients is handled by the participating hospital sites themselves. Therefore, the assignment list (code to patient) is only available to the treating hospital. At the time of recruitment, the treating physicians had no knowledge of which group the patients were assigned to.

#### Implementation {16c}

Physicians make use of the pre-randomized lists and allocate patients to the next unused slot, starting from the top. The assignment to either IG or CG is performed during consultation. After screening and handing out study information, the patient is directly allocated to IG or CG by the physician. For this purpose, the physician accesses the secure online “container,” selects the next free slot with an ID code in the randomization list, and assigns it to the patient. The extracted ID code must be entered in the alley application via installation and indicates to which group the patient has been assigned to. After this outpatient clinic appointment, patients have time to decide whether they want to participate till the next consultation (approx. 14 days before surgery). In the case of study participation, the group allocation remains; otherwise, the ID will be removed.

### Assignment of interventions: blinding

#### Who will be blinded {17a}

Neither patients nor medical stuff are blind to the intervention. Patients know whether they are IG or CG by receiving educational content or just receiving questionnaires by the app. Medical stuff will receive information on patients` medical history only from patients of the IG. Therefore, blinding during the study is not possible. However, statistical analysis is performed by a blinded analyst.

#### Procedure for unblinding if needed {17b}

Not applicable. No unblinding necessary, as trial participants and care providers are not blinded. Unblinding of the data analyst is not intended.

### Data collection and management

#### Plans for assessment and collection of outcomes {18a}

The collection of patient data is exclusively performed in digital form through the alley application. Both standardized and self-constructed questionnaires are used (Table 2). All data are registered pseudonymously in an online database.

#### Primary outcome: physical functioning

To measure the global physical functioning of the hip, the widely utilized 40-item-version of the Hip Osteoarthritis Outcome Score (HOOS) will be used. The score evaluates patient outcomes in the five subscales pain, symptoms, activity of daily living, sport and recreation function, and hip-related quality of life. The HOOS includes all Western Ontario and McMaster Universities Osteoarthritis Index (WOMAC LK 3.0) questions. The score demonstrates good construct validity and high responsiveness [22]. Physical functioning of the knee will be measured with the Knee injury and Osteoarthritis Outcome Score (KOOS). The score measures physical functioning on the same subscales and has a sufficient test-retest reliability and convergent as well as divergent construct validity [23, 35].

#### Pain

A unidimensional single-item visual analog scale (VAS-pain) is used to measure pain intensity within the last week. Pain values were measured by placing a mark on a 10 cm line representing a range between “no pain” and “worst pain.”

#### Depression and anxiety

The 21-item depression, anxiety and stress scale (DASS) is applied to screen signs of depression and anxiety. The instrument has three subscales: depression, anxiety, and stress, each with seven items. The reliability by means of Cronbach’s alpha ranged from .76 for the subscale anxiety to .88 for depression. Sensitivity (77%) and specificity (83%) are good and achieve similar results compared to established test questionnaires, e.g., Hospital Anxiety and Depression Scale [24].

#### Physiological, mental, and social health

The Patient Reported Outcomes Measurement Information System (PROMIS-29) is a research initiative to improve and standardize the measurement of self-reported physical, mental, and social health characteristics. The 29-item score surveys seven health domains (physical function, fatigue, pain interference, depressive symptoms, anxiety, ability to participate in social roles and activities, and sleep disturbance) and in addition pain in a numeric rating item ranging from 0 to 10 (no pain to worst pain). The items of the PROMIS-29 are combined into a physical and a mental health summary score with corresponding excellent reliability (.98 to .97) and good reliability [25].

#### Patient expectations

The 6-item credibility/expectancy questionnaire evaluates treatment expectancy and rationale credibility of patients. The expectancy factor of the questionnaire can predict outcome on some measures. In total, the score has a good of Cronbach’s alpha reliability of .85 [26].

#### Patient satisfaction

Patients’ Experience Questionnaire (PEQ) is a 16-item questionnaire on hospital stay. The questionnaire assesses satisfaction with physicians, nursing staff, and service. The reliability by means of Cronbach’s alpha can be considered very good ranging from .81 for service evaluation to .94 for nursing staff evaluation. Due to the good to very good correlation with scales from the Cologne patient questionnaire, the PEQ provides an initial good external validity [27].

#### Dispositional optimism and pessimism

The 10-item Life-Orientation-Test (LOT-R) survey individual differences between optimism and pessimism based on specific personality traits. The relevance of the construct of optimism has been impressively demonstrated in numerous studies. Longitudinal studies have shown positive correlations with psychological well-being, physical health, health behavior, positive recovery processes, and lower mortality. The score shows moderate reliability of .69 for optimism and.59 for pessimism by means of Cronbach’s alpha [28].

#### Other outcomes

The other parameters risk of falling, post-operative complications, knowledge about the disease and postoperative behavior (patients’ health literacy), and perceived knowledge of the patient about the disease (physician questionnaire) are evaluated with self-constructed questionnaires. The knowledge questions for the patient represents the topics of the information provided by the alley application.

### Plans to promote participant retention and complete follow-up {18b}

The electronic data collection enables time and cost reductions, as well as compliance with quality standards through automatic completion checks. Patients are reminded to fill in the questionnaires by the app. In addition, each patient receives 150 EUR after the questionnaire completion 3 months after surgery. Due to the study design and the adjuvant design of the alley intervention, a deviation from intervention protocols is not expected. Further actions to ensure a complete follow-up at all measurement time points are not intended.

### Data management {19}

All data entries of the patients are made in the alley application and stored online. The data platform is hosted in a highly secure data center in Germany (Düsseldorf, RZ equinix. DU1). Only pseudonymized data is stored with the patient’s consent. The complete study data can only be viewed by the study primary investigator (JH) for the purpose of data analysis. The electronic data collection enables automatic checks for completeness and plausibility. Input errors can be excluded due to the study design.

### Confidentiality {27}

As mentioned, only pseudonymized data is stored online with the patient’s consent. In particular, no clear names or personal data are kept online at any point. Also, no data is stored on the application or the end device itself.

The allocation of the clear names to the study data remains in the treating cooperating study sites. Once the study has been completed, the study data are transferred from the secure data center to an offline storage device (external hard drive) and kept in a vault for 10 years according to good clinical practice. The data on the servers is then deleted.

### Plans for collection, laboratory evaluation, and storage of biological specimens for genetic or molecular analysis in this trial/future use {33}

Not applicable.

### Statistical methods

#### Statistical methods for primary and secondary outcomes {20a}

A two-arm evaluation will be performed. In one arm, all patients of the “hip intervention group” and their CG are analyzed. In the other study arm, the evaluation of the “knee intervention group” and their corresponding CG is carried out. A combination of the data from the two study arms is not intended. The statistical analysis is performed separately for patients with TKA and THA by means of mean, median, standard deviation, and quantiles to initially depict the patient collectives and the primary and secondary endpoints descriptively. The Kolmogorov-Smirnov test as well as histograms and QQ-plots are applied to evaluate normal distribution of all continuous variables. Differences at baseline between IG and CG are assessed with Student’s *t*-test for independent variables in case of normally distributed data or with the Mann-Whitney *U* test in case of non-normal distribution. Categorical variables are examined by *χ*^2^ or Fisher’s exact test. If differences are observed, data risk adjustment with logistic regression is applied.

To evaluate outcome differences between the groups, delta values (change scores) are calculated in each group between the adjacent measurement time points (ti-tj, j > i) for each variable. To address our main superiority hypothesis, delta values from baseline to 3 months post-surgery of the IG and CG are statistically examined using Student’s *t*-test for independent variables. Additionally, Cohen’s *d* effect size and its 95% confidence interval will be calculated. Cohen’s *d* is a measure of the effect size for mean difference between two samples regarding interval-scaled variables. Values of *d* = 0.20 represent small, *d* = 0.50 medium, and values from *d* = 0.80 large effect [36]. In the case of non-normally distributed data, the Mann-Whitney *U* test is applied. To investigate differences at the follow-up measurement time points, further *t*-tests were performed. Because of multiple testing, an alpha error correction according to Bonferroni will be applied.

Significant differences between the several measurement times *t*_0_ to *t*_5_ are evaluated with analyses of variance with repeated measures and paired Student’s *t*-test for normally distributed data. The Friedman test and the Wilcoxon test are used in the case of non-normally distributed data.

#### Interim analyses {21b}

After 3-month post-surgery, the main analysis will be performed to address the primary research question. The analysis will be conducted by a blinded statistician and the results will be published. Main analysis will be conducted after twelve months by the same blinded statistician.

#### Methods for additional analyses (e.g., subgroup analyses) {20b}

In addition to regular statistical analysis group differences will be analyzed regarding sex, age, and secondary diseases. If significant differences are found, risk adjustment will be conducted by logistic regression analysis.

#### Methods in analysis to handle protocol non-adherence and any statistical methods to handle missing data {20c}

The allocation of patients to the IG and KG is realized via an individual ID, which can only be assigned once. Once the patient enters the ID received from the attending physician into the alley application, the patient is clearly assigned to one of the two groups. Accordingly, protocol errors regarding randomization are not to be expected. Further, it is not possible to verify whether the patients of the IG are reading the provided digital information. Feedback on the usefulness of the application is only provided indirectly by physicians and patients themselves during the survey.

As this trial uses an online survey, missing data within one questionnaire is not possible. If a survey is not completed at a measurement point, the existing data will be used for the evaluation. Generally, an intention-to-treat (ITT) analysis as well as a per protocol (PP)-analysis will be performed. However, the PP analysis is used exclusively to validate the ITT analysis. To address our research question, the main analyses are carried out exclusively by means of ITT.

#### Plans to give access to the full protocol, participant-level data, and statistical code {31c}

Not applicable; no plans to give external access to the full protocol, participant level-data, or statistical code.

### Oversight and monitoring

#### Composition of the coordinating center and trial steering committee {5d}

Not applicable. The study is coordinated by the Department of Orthopaedics at the University Medical Center Hamburg-Eppendorf. There is a research fellow who is mainly responsible for the implementation of the study, but no specific trial steering committee has been established.

#### Composition of the data monitoring committee, its role and reporting structure {21a}

Due to the direct input of data and the central digital storage with direct access by the primary investigator, a data monitoring committee is not necessary. There will be no external data and safety monitoring board. Data and safety monitoring will be the responsibility of the principal and associate investigators.

#### Adverse event reporting and harms {22}

The alley application does not involve any risk for the users beyond those associated with standard care. Patients in the IG merely receive detailed information and complete questionnaires. Patients in the CG complete questionnaires only. In both groups, the alley application is given as a supplement on top to the standard treatment (TJA). In addition, the application is approved as a “class 1 medical device.” A systematic literature search, which was conducted in the process of obtaining approval as a medical device, indicated that studies with comparable applications do not provide any indication of hazards or harm [37, 38, 39, 40, 41].

Participation in this trial will not entail additional risks beyond those associated with standard care.

#### Frequency and plans for auditing trial conduct {23}

After training the individual trial sites in the use of the alley application and patient recruitment, no further structured audits are planned. This approach is justified as after recruitment, no further interventions need to be conducted by the medical staff at the hospital sites. The app operates autonomously on the patient’s mobile device.

#### Plans for communicating important protocol amendments to relevant parties (e.g., trial participants, ethical committees) {25}

There are no specific risks in relation to the study itself. In case of important unexpected protocol modifications, all multi-center study sites will be informed, recruitment will be temporarily discontinued and the local ethics committee will be informed by amendment. Concurrently, the participating patients will be informed in writing about the changes.

#### Dissemination plans {31a}

The app will be available in App stores. The results of the study will be used to optimize app features and will be published in peer reviewed journals.

## Discussion

Improvement of care for patients with TJA is a subject of ongoing discussion [10]. Over time, different digital approaches have been developed to address this issue. Digital technologies aim for improvement by targeting different aspects of care, such as patient education, structural improvements of pre- and post-operative management, physical training programs, and enhancing health-related quality of life [e.g., [16, 33]. The use of eHealth tools enables medical advice and support independent of actual contact hours and may lead to cost savings [19, 20], as has been indicated in systematic reviews [21]. Next to general economic effects, it was shown that patients with hip or knee osteoarthritis benefit from a targeted educational and self-directed training programs by significantly reduced pain and an improvement in quality of life [42]. Further studies suggest improved treatment results and reduced length of inpatient stay [14, 15]. A Cochrane review summarizes the effects of pre-surgical education of patients with hip and knee arthroplasty to be beneficial regarding cost efficiency, unwanted results, pain, and physical functioning [13].

Digital solutions offer a promising opportunity to provide pre-operative education to achieve the above-mentioned improvements. In line with these findings, effects of the digital alley application are likely to be found in various areas. With the present multi-center study, the effectiveness of the adjuvant application is compared with a usual care therapy CG (gold standard treatment group). Alley’s approach is novel since the app combines an intelligent companion and information platform along the treatment pathway with direct and continuous feedback from the patients to the therapeutic treatment team due to standardized questionnaires implemented in the application. By offering a dashboard that includes patient entered data to the medical stuff, the medical consultation hours can be tailored to individual patient needs, allowing more efficient medical consultations in terms of both time/costs and quality. Doctors and caregivers can use the alley dashboard in various situations, e.g., digital anamnesis support, early identification of risks and complications, and follow-up of treatment success. Intensity of the use of the dashboard is not measured or evaluated as part of the study. In comparison, the applying patents receive emotional, content-related, and administrative support throughout the treatment pathway. Accordingly, it can be assumed that patients received higher satisfaction with treatment, experienced increased empowerment, and increased quality of life.

## Strengths and limitations

The present study will have clear strengths and weaknesses. Methodologically, the study is designed as a prospective, randomized, controlled, multi-center trial with clear inclusion and exclusion criteria. Although the basic conditions of the patients are similar in all clinics, it cannot be excluded that clinic-internal processes or procedures influence the change in outcome (e.g., different surgical accesses, special pain management within a clinic, etc.). Since the study examined the effectiveness under everyday conditions, it could not be controlled whether patients in the IG read the patient-educational information provided by the “alley ortho companion” app or whether patients in the CG did consume additional information from the Internet or brochures in preparation for TJA. However, this type of study design increases the external validity, which improves the generalizability of the study results. Concurrently, it should be noted that there are certain limitations regarding the transferability of the results to the general target group of the application. The external validity could be distorted by the incentive of 150 EUR per patient. We cannot exclude the possibility that the patients have engaged with the app particularly intensively for this reason. Nevertheless, we believe that differences in the HOOS and KOOS score between the IG and CG can still be directly attributed to the intervention due to the randomized controlled trial design. Another limitation is that the use of a digital application requires the ownership of a mobile device, the ability to handle the device, and the ability to handle the app itself. Due to the advanced age of the patients with osteoarthritis, some individuals could be systematically excluded from the effects of the intervention. For these patients, the most important core information from the alley application could be provided to as written handout or brochure. To systematically examine this potential third study arm, the sample size would have had to be highly increased, which was not possible for logistical and economic reasons in the current design.

## Conclusion

Overall, the study aims to improve the understanding of the benefits of eHealth applications in the treatment of older people with knee or hip arthroplasty, which has a positive impact on society and the health economy. The results could provide beneficial effects for study participants. Patients will be able to monitor their symptoms and recognize changes at an early stage, which may be cause for medical consultation.

## Trial status

In July 2021, the first patients were included in selected study sites to evaluate the proposed recruitment management and functionality of the application according to our ethical approved study protocol (ver. 2 from 12 May 2021). Active patient recruitment by all study sites is conducted from 01 September 2021 till 30 February 2022, followed by a maximum of 12-month follow-up.

## Data Availability

The study data are not available to the public. Study data can only be viewed and retrieved by the study primary investigator (JH).

## Abbreviations

DASS: Depression, anxiety and stress scale
CG: Control group
eHealth: Electronic Health
HOOS: Hip Osteoarthritis Outcome Score
ICHOM: International Consortium for Health Outcomes Measurement
ID: Identifier
IG: Intervention group
KOOS: Knee Osteoarthritis Outcome Score
LOT-R: Life-Orientation-Test
THA: Total hip arthroplasty
TJA: Total joint arthroplasty
TKA: Total knee arthroplasty
OA: Osteoarthritis
PEQ: Adapted Patients’ Experience Questionnaire
PROMIS®-29: Patient Reported Outcomes Measurement Information System
QQ-plot: Quantile-Quantile-Plots
VAS: Visual analog scale
WOMAC: Western Ontario and McMaster Universities Osteoarthritis Index
WHO: World Health Organization
